# Molecular modelling insights into a physiologically favourable approach to eicosanoid biosynthesis inhibition through novel thieno[2,3-*b*]pyridine derivatives

**DOI:** 10.1080/14756366.2018.1457657

**Published:** 2018-04-13

**Authors:** Mosaad S. Mohamed, Yara E. Mansour, Hatem K. Amin, Moustafa E. El-Araby

**Affiliations:** a Department of Pharmaceutical Organic Chemistry, Faculty of Pharmacy, Helwan University, Cairo, Egypt;; b Department of Biochemistry and Molecular Biology, Faculty of Pharmacy, Helwan University, Cairo, Egypt

**Keywords:** Anti-inflammatory, COX-2, 5-LOX, COX-1, thieno[2,3-b]pyridine

## Abstract

In this research, we exploited derivatives of thieno[2,3-*b*]pyridine as dual inhibitors of the key enzymes in eicosanoid biosynthesis, cyclooxygenase (COX, subtypes 1 and 2) and 5-lipoxygensase (5-LOX). Testing these compounds in a rat paw oedema model revealed potency higher than ibuprofen. The most active compounds **7a**, **7b**, **8b**, and **8c** were screened against COX-1/2 and 5-LOX enzymes. Compound **7a** was the most powerful inhibitor of 5-LOX with IC_50_ = 0.15 µM, while its *p-*chloro analogue **7b** was more active against COX-2 (IC_50_ = 7.5 µM). The less desirable target COX-1 was inhibited more potently by **8c** with IC_50_ = 7.7 µM. Surflex docking programme predicted that the more stable *anti-* conformer of compound (**7a**) formed a favourable complex with the active site of 5-LOX but not COX-1. This is in contrast to the binding mode of **8c**, which resembles the *syn*-conformer of series **7** and binds favourably to COX-1.

## Introduction

Eicosanoids are arachidonic acid (AA) metabolites that comprise prostaglandins (PGs) and leukotrienes (LTs), the principal inflammatory mediators in osteoarthritis (OA)^1^^,^^2^. PGs and LTs play key roles in triggering the failure of innate homeostasis of chondrocytes that contribute to pain, swelling, joint damage, and other symptoms of OA^3^^,^^4^. PGs’ biosynthesis starts when cyclooxygenases (COXs) catalyse two reactions, first the bis-peroxidation of AA to PGG2 and second, the reduction of the acyclic peroxide group in PGG2 to form the key precursor PGH2. PGH2 metabolises, according to elicited biological needs, to responsive PGs under catalysis of various prostaglandin synthases^5^.

**Figure 1.**
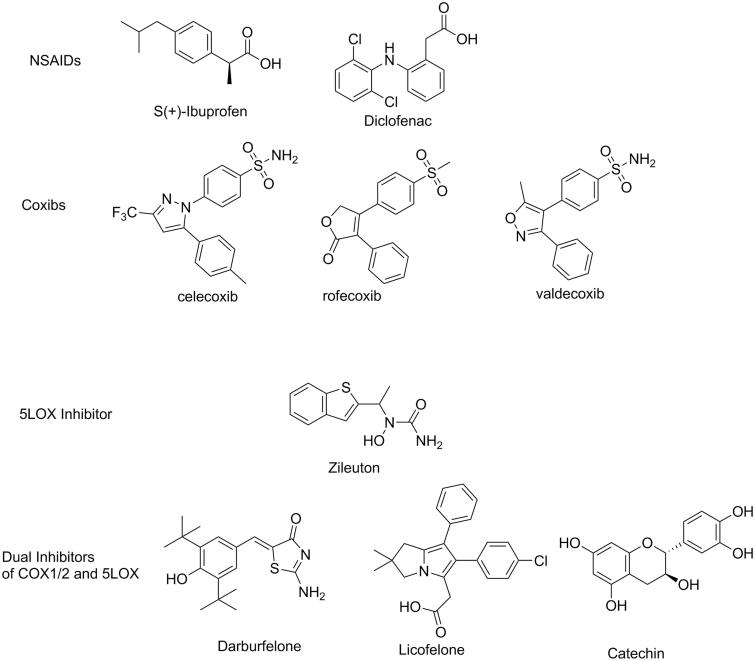
Structures of reported inhibitors of arachidonic acid biosynthesis.

The “mostly” inducible subtype cyclooxygenase-2 (COX-2) is greatly upregulated during the inflammatory processes while the constitutive subtype COX-1 is “mostly” assigned to the regulation of PGs in healthy individuals^3^. In 1990s, the approach of selective inhibition of COX-2 by coxibs was sought as the imminent strategy to control OA, taking the advantage of less side effects than the classic non-steroidal anti-inflammatory drugs (NSAIDs, Figure 1)^6^. NSAIDs (non-selective inhibitors of COX 1&2), though widely used as analgesics and anti-inflammatory drugs to date, are frequently implicated in a variety of adverse actions. Patients under NSAIDs frequently report GI inflammation, bleeding, ulcers, in addition to hepatic problems, renal toxicities, and others^2^. However, only few years after the first coxib approval, rofecoxib, and valdecoxib (Figure 1) were withdrawn from world markets subsequent to findings of links to increased cardiovascular events such as atherosclerosis and infarction^7^^,^^8^. Other coxibs and some NSAIDs had to add warnings to their labels and limitation of uses in certain patients with risks of cardiovascular complications^9^. Before withdrawals, some valuable studies placed selective COX-2 inhibitors under scrutiny as evidences accumulated about COX-1 and COX-2 overlapping functions. COX-1’s role was proved not to be limited to “housekeeping” as it was widely claimed in literature^10^. COX-1 was found to contribute considerably to some inflammatory responses^11^ leading to decreased efficacy of some COX-2 selective inhibitors unless they exert a minimum of COX-1 inhibition at the therapeutic dose^12^. Moreover, COX-2 is constitutively expressed in certain tissues to catalyse the biosynthesis of some beneficial PGs such as PGI_2_, a critically needed prostaglandin for homeostasis of the cardiovascular system^13^. Therefore, the clinical use of COX1/2 inhibitors will remain associated with some undesirable effects regardless being a selective coxib or a non-selective NSAID^14^. Moreover, shutting down the COX1/2 pathway causes a shunting of AA to the other proinflammatory route, the lipoxygenase (LOX) pathway. This possibly results in decreased efficacy^15^ as well as aggravation of GI side effect of NSAIDs^16^. Encoding gene of 5-lipooxygensase (5-LOX), the key enzyme for biosynthesis of the inflammatory mediators LTs, is almost undetectable in healthy individual^17^ but activated during various inflammatory events^18^. Inhibitors of 5-LOX (e.g. Zileuton, Figure 1) are compassionately prescribed for controlling some inflammatory disorders such as bronchial asthma^19^, but it is much less efficacious in OA^20^ regardless the proven involvement of 5-LOX in arthritic progression^21^^,^^22^.

Based on undisputable findings, dual inhibitors for both COX1/2 and 5-LOX pathways should provide superior efficacies in controlling inflammation and pain in patients suffering from OA with less adverse effects^23^. For instance, dual COX1/2–5LOX inhibitors demonstrated excellent analgesic and anti-inflammatory activities with lower gastric irritation, bleeding and ulcerogenic side effects. In addition, dual inhibition of COX1/2 and 5-LOX provided cardioprotective activity in a noteworthy contrast to COX-2 inhibitors^24^. Efforts for developing clinically useful dual COX1/2–5LOX inhibitors are still active area in drug discovery, since no approval has been granted yet for the first drug in this class^2^. Licofelone, the first clinically studied dual inhibitor has completed phase III clinical trials but it is not clear why it is not marketed yet^2^. The potential benefits of dual COX1/2–5LOX inhibitors extend beyond OA. For instance, they demonstrated anticonvulsant^25^ as well as anticancer activities^26^^,^^27^.

**Figure 2.**
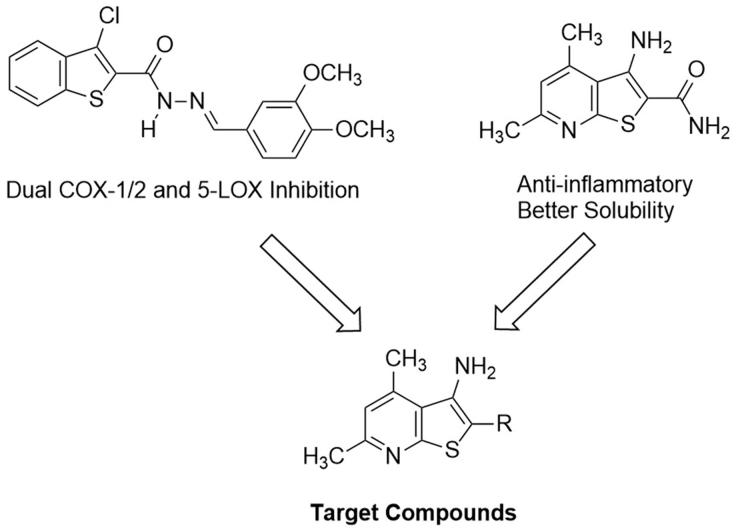
Approach to develop target compounds with dual COX1/2 and 5-LOX inhibition from previously reported compounds.

In our quest to finding novel anti-inflammatory agents, we relied on a screening model which primarily started with measuring the *in vivo* anti-inflammatory activities^28–30^. On the other hand, we sought 3-aminothieno[2,3-*b*]pyridine as promising molecular entities to develop novel anti-inflammatory agents with possible dual inhibitory mechanism against COX1/2 and 5-LOX. This hetero-bicyclic scaffold has been studied for its versatile activities against inflammatory disorders, albeit for other subcellular targets^31–33^. Thienopyridine derivatives have also been reported for their potent anti-proliferative activities^34^^,^^35^. Moreover, our target thieno[2,3-*b*]pyridine is a close congener to benzothiophene, a nucleus recently tested for inhibition of COX1/2 and 5-LOX^36^ (Figure 2).

## Experimental

### Chemistry

#### General methods

All melting points were uncorrected and measured using Electro-thermal IA 9100 apparatus (Shimadzu, Japan); IR spectra were recorded as potassium bromide pellets on a Perkin-Elmer 1650 spectrophotometer (USA), Faculty of Science, Cairo University, Cairo, Egypt. ^1^H-NMR spectra were determined on a Varian Mercury (500 MHz) spectrometer (Varian UK) and chemical shifts were expressed as ppm against TMS as an internal reference (Faculty of Science, CairoUniversity, Cairo, Egypt). Mass spectra were recorded on 70 eV (EI Ms-QP 1000 EX, Shimadzu, Japan), Faculty of Science, CairoUniversity, Cairo, Egypt. Microanalyses were operated using Vario, Elmentar apparatus (Shimadzu, Japan), Organic Microanalysis Unit, Faculty of Science, Cairo University, Cairo, Egypt. Column Chromatography was performed on (Merck) Silica gel 60 (particle size 0.06–0.20 mm).

#### Synthesis

##### 1,2-Dihydro-4,6-dimethyl-2-thioxopyridine-3-carbonitrile (1)

A mixture of 4,6-dimethyl-3-cyano-2-chloropyridine (1.66 g, 0.01 mol) and thiourea (1.52 g, 0.02 mol) was refluxed in dry ethanol (20 ml) for 6 h^37^. Then evaporated under reduced pressure and the residue was recrystallised from methanol to give **1**. Yield 68%, m.p. 183 °C (Lit. m.p. 180 °C^24^).

##### General procedure for preparation of compounds (2a–c)

A mixture of the pyridinethione **1** (1.64 g, 0.01 mol), chloroacetic acid (2.8 g, 0.03 mol), appropriate aldehyde (0.05 mol), and fused sodium acetate (2.0 g) in acetic acid/acetic anhydride (30 ml, 1:1) was heated under reflux for 8 h and left to cool. The solid formed was filtered off and washed with water and recrystallised from ethanol/H_2_O to afford **2a**–**c.**

##### (±) 3-*Benzylidene*-3, 8a-dihydro-5,7-dimethyl-2-oxo-2*H*-thiazolo[3,2-*a*]pyridine-8-carbonitrile (2a)

Yield: 77%; m.p.: 105–107 °C; IR (KBr) υ (cm^−1^): 1725 (C=O), 2256 (C≡N). MS (EI) *m*/*z*: 294 [M‏^+^] (85%). ^1^H NMR (DMSO-d_6_, 500 MHz) δ (ppm): 2.41 (s, 3H, CH_3_), 2.75 (s, 3H, CH_3_), 5.4 (s, 1H, CH), 6.5 (s, 1H, pyr-H), 6.9 (s, H, =CH), 7.1–7.6 (m, 5H, Ar-H).; Anal. Calcd. for C_17_H_14_N_2_OS (294.08): C, 69.36; H, 4.79; N, 9.52%. Found: C, 69.67; H, 4.42; N, 9.79%.

##### (±) 3-*Furan-2-ylmethylene*-3,8a-dihydro-5,7-dimethyl-2-oxo-2*H*-thiazolo[3,2-*a*]pyridine-8-carbonitrile (2b)

Yield: 69%; m.p.: 100–102 °C; IR (KBr) υ (cm^−1^): 1730 (C=O), 2220 (C≡N). MS (EI) *m*/*z*: 284 [M‏^+^] (85%). ^1^H NMR (DMSO-d_6_, 500 MHz) δ (ppm): 2.12 (s, 3H, CH_3_), 2.3 (s, 3H, CH_3_), 5.5 (s, 1H, CH), 6.4 (s, 1H, pyr-H), 7.0 (s, H, =CH), 7.1–7.4 (m, 3H, Ar-H).; Anal. Calcd. for C_15_H_12_N_2_O_2_S (284.06): C, 63.36; H, 4.25; N, 9.85%. Found: C, 63.14; H, 4.59; N, 9.61%.

##### (±)3-*(3-Phenyl-allylidene)*-3,8a-dihydro-5,7-dimethyl-2-oxo-2*H*-thiazolo[3,2-*a*]pyridine-8-carbonitrile (2c)

Yield: 76%; m.p.: 80–82 °C; IR (KBr) υ (cm^−1^): 1580 (C = C), 1750 (C=O), 2260 (C≡N). MS (EI) *m*/*z*: 320 [M‏^+^] (85%). ^1^H NMR (DMSO-d_6_, 500 MHz) δ (ppm): 2.2 (s, 3H, CH_3_), 2.4 (s, 3H, CH_3_), 5.4 (s, 1H, CH), 6.2 (s, 1H, pyr-H), 6.5 (s, H, =CH), 6.7 (s, H, =CH), 7.1 (s, H, =CH), 7.3–7.6 (m, 5H, Ar-H). Anal. Calcd. for C_19_H_16_N_2_OS (320.1): C, 71.22; H, 5.03; N, 8.74%. Found: C, 71.53; H, 5.28; N, 8.45%.

##### General procedure for preparation of compounds 3a–b

To an ethanolic sodium hydroxide solution [sodium hydroxide (0.01 mol) in ethanol (20 ml)] pyridinethione **1** (1.64 g, 0.01 mol) was added, with stirring for 30 min, and then the appropriate alkyl halide (0.02 mol) was added drop-wise with vigorous shaking. The reaction mixture was stirred for 2 h at r.t., poured onto cold water (100 ml) and neutralised with dilute HCl. The solid precipitate was filtered off and washed with water and recrystallised from methanol to afford **3a**–**b**. The reported compound **3b** was prepared in 70% yield and m.p. 93 °C (Lit. m.p. 90 °C)^37^.

##### *S*-Methyl-4, 6-dimethyl-2-thiopyridine-3-carbonitrile (3a)

Yield: 72%; m.p.: 80–82 °C; IR (KBr) υ (cm^−1^): 2218 (C≡N). MS (EI) *m*/*z*: 178 [M‏^+^] (91%). ^1^H NMR (DMSO-d_6_, 500 MHz) δ (ppm): 2.59 (s, 3H, CH_3_), 2.65 (s, 3H, CH_3_), 3.96 (s, 3H, S-CH_3_), 6.9 (s, 1H, pyr-H). Anal. Calcd. for C_9_H_10_N_2_S (178.25): C, 60.64; H, 5.65; N, 15.72%. Found: C, 60.41; H, 5.84; N, 15.96%.

##### General procedure for preparation of compounds 4a–c

An excess of aqueous 37% fomaldehyde (5–6 ml) was added to a suspension of pyridinethione **1** (1.64 g, 0.01 mol) in EtOH (15 ml). The mixture was heated with stirring to complete dissolution and then the appropriate primary amine (0.01 mol) was added in one portion. The resulting mixture was refluxed with vigorous stirring for 3 min and then stirred at 20 °C for 4 h. The precipitate formed was filtered off and crystallised from ethanol to afford **4a**–**c.**

##### (±)3-Aryl-2,3,4,9a-tetrahydro-6,8-dimethylpyrido[2,1-*b*][1,3,5]thiadiazine-9-carbonitrile (4a)

Yield: 53%; m.p.: 180–182 °C; IR (KBr) υ (cm^−1^): 2230 (C≡N). MS (EI) *m*/*z*: 283 [M‏^+^] (69%). ^1^H NMR (DMSO-d_6_, 500 MHz) δ (ppm): 2.30 (s, 3H, CH_3_), 2.58 (s, 3H, CH_3_), 4.5 (s, 2H, S-CH_2_), 4.9 (s, 2H, N-CH_2_), 5.2 (s, 1H, CH), 6.8 (s, 1H, pyr-H), 6.9–7.5 (m, 5H, Ar-H). Anal. Calcd. for C_16_H_17_N_3_S (283): C, 67.81; H, 6.05; N, 14.83%. Found: C, 67.65; H, 6.31; N, 14.62%.

##### (±)3-Aryl-2,3,4,9a-tetrahydro-6,8-dimethylpyrido[2,1-*b*][1,3,5]thiadiazine-9-carbonitrile (4b)

Yield: 68%; m.p.: 115–117 °C; IR (KBr) υ (cm^−1^): 2235 (C≡N). MS (EI) *m*/*z*: 297 [M‏^+^] (79%). ^1^H NMR (DMSO-d_6_, 500 MHz) δ (ppm): 2.1 (s, 3H, CH_3_), 2.3 (s, 3H, CH_3_), 2.8 (s, 3H, CH_3_), 4.6 (s, 2H, S-CH_2_), 4.8 (s, 2H, N-CH_2_), 5.1 (s, 1H, CH), 6.2 (s, 1H, pyr-H), 7.0–7.5 (m, 4H, Ar-H). Anal. Calcd. for C_17_H_19_N_3_S (297): C, 68.65; H, 6.44; N, 14.13%. Found: C, 68.31; H, 6.25; N, 14.39%.

##### (±)3-Aryl-2,3,4,9a-tetrahydro-6,8-dimethylpyrido[2,1-*b*][1,3,5]thiadiazine-9-carbonitrile (4c)

Yield: 59%; m.p.: 125–127 °C; IR (KBr) υ (cm^−1^): 2210 (C≡N). MS (EI) *m*/*z*: 297 [M‏^+^] (72%). ^1^H NMR (DMSO-d_6_, 500 MHz) δ (ppm): 2.30 (s, 3H, CH_3_), 2.58 (s, 3H, CH_3_), 4.5 (s, 2H, S-CH_2_), 4.7 (s, 2H, CH_2_), 5.2 (s, 2H, N-CH_2_), 5.9 (s, 1H, CH), 6.8 (s, 1H, pyr-H), 7.2–7.6 (m, 5H, Ar-H). Anal. Calcd. for C_17_H_19_N_3_S (297): C, 68.65; H, 6.44; N, 14.13%. Found: C, 68.88; H, 6.71; N, 14.00%.

##### Ethyl-3-amino-4,6-dimethylthieno[2,3-*b*]pyridine-2-carboxylate (5)

This compound was prepared by two methods.

*Method 1*: A mixture of pyridinethione **1** (1.64 g, 0.01 mol) and ethyl bromoacetate (3.34 g, 0.02 mol) in dry ethanol (50 ml) containing sodium metal (1 g) was stirred for 2 h, and then was refluxed with stirring for 4 h. The reaction mixture was cooled and poured into ice water. The solid formed was filtered off, dried, and recrystallised from methanol to afford **5**.

*Method 2*: A mixture of ethyl 2-(3-cyano-4,6-dimethylpyridin-2-ylthio)acetate **3b** (2.5 g, 0.01 mol) and dry ethanol (50 ml) containing sodium ethoxide (2%) was refluxed with stirring for 4 h. The reaction mixture was cooled and poured into ice water. The solid formed was filtered off, dried, and recrystallised from ethanol to afford **5**.

Yield 70%, m.p. 138 °C (Lit. m.p. 135 °C^24^).

##### 3-Amino-4,6-dimethylthieno[2,3-*b*]pyridine-2-carbazide (6)

A mixture of thienopyridine-2-carboxylate **5** (2.5 g, 0.01 mol) and hydrazine hydrate (0.05 mol, 99%) in absolute ethanol (15 ml); was heated under reflux for 6 h. The reaction mixture was concentrated, cooled, and the precipitate formed was filtered and crystallised from ethanol to afford **6.** Yield 70%, m.p. 200 °C (Lit. m.p. 198 °C)^37^.

##### General procedure for preparation of compounds 7a–c

A mixture of thienopyridine-2-carbazide (2.36 g, 0.01 mol) and the appropriate aldehyde (0.02 mol) in pyridine:ethanol (15:20 ml) was heated under reflux for 2 h. The reaction mixture was cooled and poured into ice water. The solid formed was filtered off, dried, and crystallised from the acetic acid to afford **7a**–**c**.

##### 3-Amino-4,6-dimethyl-thieno [2,3-*b*] pyridine-2-carboxylic acid benzylidene-hyrazide (7a)

Yield: 65%; m.p.: 250–252 °C; IR (KBr) υ (cm^−1^): 3100, 3260, 3310 (NH)(NH_2_), 1690 (C=O), 1230 (C=N). MS (EI) *m*/*z*: 324 [M^+^] (76%). ^1^H NMR (DMSO-d_6_, 500 MHz) δ (ppm): 2.32 (s, 3H, CH_3_), 2.71 (s, 3H, CH3), 7.0 (s, 1H, pyr-H), 6.9 (s, 2H, NH_2_, D_2_O exchangeable), 7.3–7.6 (dd, 5H, Ar-H), 8.2 (s, 1H, NH, D_2_O exchangeable), 8.5 (s, 1H, CH). Anal. Calcd. for C_17_H_16_N_4_OS (324): C, 62.94; H, 4.97; N, 17.27%. Found: C, 62.97; H, 4.62; N, 17.62%.

##### 3-Amino-4,6-dimethyl-thieno [2,3-*b*] pyridine-2-carboxylic acid (4-chloro-benzylidene)-hyrazide (7b)

Yield: 72%; m.p.: 280–282 °C; IR (KBr) υ (cm^−1^): 3150, 3290, 3310 (NH)(NH_2_), 1685 (C=O), 1245 (C=N). MS (EI) *m*/*z*: 358 [M^+^] ^35^Cl (66%), 360 [M + 2] ^37^Cl (22%), ^1^H NMR (DMSO-d_6_, 500 MHz) δ (ppm): 2.45 (s, 3H, CH_3_), 2.62 (s, 3H, CH_3_), 6.9 (s, 1H, pyr-H), 7.0 (s, 2H, NH_2_, D_2_O exchangeable), 7.2–7.5 (dd, 4H, Ar-H), 8.3 (s, 1H, NH, D_2_O exchangeable), 8.6 (s, 1H, CH). Anal. Calcd. for C_17_H_15_ClN_4_OS (358): C, 56.90; H, 4.21; N, 15.61%. Found: C, 56.46; H, 4.49; N, 15.93%.

##### 3-Amino-4, 6-dimethyl-thieno [2,3-*b*] pyridine-2-carboxylic acid (4-methoxy-benzylidene)-hyrazide (7c)

Yield: 74%; m.p.: 270–272 °C; IR (KBr) υ (cm^−1^): 3180, 3240, 3320 (NH) (NH_2_), 1680 (C=O), 1220 (C=N), 1150 (C–O). MS (EI) *m*/*z*: 354 [M^+^](58%). ^1^H NMR (DMSO-d_6_, 500 MHz) δ (ppm): 2.32 (s, 3H, CH_3_), 2.61 (s, 3H, CH_3_), 3.5 (s, 3H, CH_3_), 6.8 (s, 1H, pyr-H), 7.0 (s, 2H, NH_2_, D_2_O exchangeable), 7.2–7.6 (dd, 4H, Ar-H), 8.0 (s, 1H, NH, D_2_O exchangeable), 8.6 (s, 1H, CH). Anal. Calcd. for C_18_H_18_N_4_O_2_S (354): C, 61.00; H, 5.12; N, 15.81%. Found: C, 61.33; H, 5.45; N, 15.56%.

##### General procedure for preparation of compounds 8a–c

To an ice-cooled solution of the corresponding thienopyridine-2-carboxylic acid aryldine-hydrazide **7a**–**c** (0.01 mol) in AcOH (20 ml) and conc. HCl (3 ml) a cold solution of sodium nitrite (0.23 g in 5 ml of water) was added drop-wise over 5 min. The solution was stirred at room temperature for 3 h at 0–5 °C; where a solid was formed, collected by filtration, dried, and crystallised from dioxane to afford **8a**–**c**.

##### 7,9-Dimethyl-3-[(phenylmethylene)amino]pyrido[3′,2′:4,5]thieno[3,2-*d*][1,2,3]triazin-4(3H)-one (8a)

Yield: 72%; m.p.: 300–302 °C; IR (KBr) υ (cm^−1^): 1690 (C=O), 1250 (C=N). MS (EI) *m*/*z*: 335 [M^+^] (45%), ^1^H NMR (DMSO-d_6_, 500 MHz) δ (ppm): 2.35 (s, 3H, CH_3_), 2.75 (s, 3H, CH_3_), 6.8 (s, 1H, pyr-H), 7.3–7.6 (m, 5H, Ar-H), 8.2 (s, 1H, CH). Anal. Calcd. for C_17_H_13_N_5_OS (335): C, 60.88; H, 3.91; N, 20.88%. Found: C, 60.47; H, 3.64; N, 20.57%.

##### 7,9-Dimethyl-3-[(4-chloro-phenyl-methylene)amino]pyrido[3ˈ,2ˈ:4,5] thieno [3,2-*d*][1,2,3]triazin-4(3H)-one (8b)

Yield: 74%; m.p.: 255–257 °C; IR (KBr) υ (cm^−1^): 1685 (C=O), 1270 (C=N). MS (EI) *m*/*z*: 369 [M^+^] ^35^Cl (60%), 371 [M + 2] ^37^Cl (20%). ^1^H NMR (DMSO-d_6_, 500 MHz) δ (ppm): 2.45 (s, 3H, CH_3_), 2.62 (s, 3H, CH_3_), 6.9 (s, 1H, pyr-H), 7.2–7.5 (dd, 4H, Ar-H), 8.3 (s, 1H, CH). Anal. Calcd. for C_17_H_12_ClN_5_OS (369): C, 55.21; H, 3.27; N, 18.94%. Found: C, 55.49; H, 3.51; N, 18.72%.

##### 7,9-Dimethyl-3-[(4-methoxy-phenyl-methylene)amino]pyrido[3ˈ,2ˈ:4,5]thieno [3,2-*d*][1,2,3]triazin-4(3H)-one (8c)

Yield: 65%; m.p.: 240–242 °C; IR (KBr) υ (cm^−1^): 1680 (C=O), 1240 (C=N), 1150 (C–O). MS (EI) *m*/*z*: 365 [M^+^] (56%), ^1^H NMR (DMSO-d_6_, 500 MHz) δ (ppm): 2.32 (s, 3H, CH_3_), 2.61 (s, 3H, CH_3_), 3.5 (s, 3H, CH_3_), 7.0 (s, 1H, pyr-H), 7.2–7.6 (dd, 4H, Ar-H), 8.6 (s, 1H, CH). Anal. Calcd. for C_18_H_15_N_5_O_2_S (365): C, 59.16; H, 4.14; N, 19.17%. Found: C, 59.42; H, 4.41; N, 19.54%.

##### General procedure for preparation of compounds 9a–b

A mixture of thienopyridine-2-carbazide **6** (2.36 g, 0.01 mol) and ethyl acetoacetate (1.3 g, 0.01 mol) or acetylacetone (1 g, 0.01 mol) in glycial acetic acid (30 ml) was heated under reflux for 5 h. The excess solvent was evaporated under reduced pressure to one-third of the solution and the reaction mixture was allowed to cool. The precipitate formed was collected and crystallised from the acetic acid to afford **9a** and **9b**.

##### 3-Amino-2-[carbonyl (3-methyl-4,5-dihydro-5H-pyrazol-5-on-1-yl)]-4,6-dimethylthieno [2,3-*b*] pyridine (9a)

Yield: 59%; m.p.: 140–142 °C; IR (KBr) υ (cm^−1^): 3425, 3265 (NH_2_), 1686 (C=O). MS (EI) *m*/*z*: 302 [M^+^‏] (71%), ^1^H NMR (DMSO-d_6_, 500 MHz) δ (ppm): 2.00 (s, 3H, CH_3_), 2.29 (s, 3H, CH_3_), 2.65 (s, 3H, CH_3_), 2.9 (s, 2H,CH_2_), 5.58 (s,2H,NH_2_, D_2_O exchangeable), 6.9 (s, 1H, pyr-H).; Anal. Calcd. for C_14_H_14_N_4_O_2_S (302.35): C, 55.61; H, 4.67; N, 18.53%. Found: C, 55.39; H, 4.90; N, 18.81%.

##### 3-Amino-2-[carbonyl (3,5-dimethyl-pyrazol-1-yl)]-4,6-dimethylthieno[2,3-*b*] pyridine (9b)

Yield: 67%; m.p.: 150–152 °C; IR (KBr) υ (cm^−1^): 3425, 3265 (NH_2_), 1686 (C=O). MS (EI) *m*/*z*: 300 [M^+^‏] (62%), ^1^H NMR (DMSO-d_6_, 500 MHz) δ (ppm): 2.38 (s, 3H, CH_3_), 2.65 (s, 3H, CH_3_), 2.79 (s, 6H, two CH_3_), 5.2 (s, 2H, NH_2_, D_2_O exchangeable), 7.1 (s, 1H, pyr-H), 8.2 (s, 1H,4H-pyrazole). Anal. Calcd. for C_15_H_16_N_4_OS (300.38): C, 59.98; H, 5.37; N, 18.65%. Found: C, 59.68; H, 5.68; N, 18.40%.

##### General procedure for preparation of compounds 10a–b

A mixture of thienopyridine-2-carbazide **6** (0.01 mol) in formic acid (15 ml) or acetic anhydride (15 ml) was heated under reflux for 6 h. The excess solvent was evaporated *in vacuo* (to one-third of the solution) and cooled; the solid was collected by filtration, dried, and crystallised from acetic acid to afford **10a**–**b**.

##### 7,9-Dimethyl-3-fomylaminopyrido [3',2':4,5]thieno[3,2-*d*]pyrimidin-4(3H)-one (10a)

Yield: 48%; m.p.: 220–222 °C; IR (KBr) υ (cm^−1^): 1715, 1760 (two C=O). MS (EI) *m*/*z*: 274 [M^+^] (71%), ^1^H NMR (DMSO-d_6_, 500 MHz) δ (ppm): 2.4 (s, 3H, CH_3_), 2.8 (s, 3H, CH_3_), 7.1 (s, 1H, pyr-H), 7.9 (s, 1H, CH pyrimidine), 8.5 (s, 1H, H-C=O), 9.5 (s, 1H, NH). Anal. Calcd. for C_12_H_10_N_4_O_2_S (274.3): C, 52.54; H, 3.67; N, 20.43%. Found: C, 52.81; H, 3.41; N, 20.69%.

##### 3-Diacetylamino-2,7,9-trimethylpyrido [3',2':4,5]thieno[3,2-*d*]pyrimidin-4(3*H*)-one (10b)

Yield: 65%; m.p.: 133–135 °C; IR (KBr) υ (cm^−1^): 1720, 1745, 1780 (three C=O). MS (EI) *m*/*z*: 344 [M^+^] (65%), ^1^H NMR (DMSO-d_6_, 500 MHz) δ (ppm): 1.9 (s, 3H, CH_3_), 2.2 (s, 3H, CH_3_), 2.5 (s, 3H, CH_3_), 2.7 (s, 6H, 2CH_3_), 7.2 (s, 1H, pyr-H). Anal. Calcd. for C_16_H_16_N_4_O_3_S (344.09): C, 55.80; H, 4.68; N, 16.27%. Found: C, 56.00; H, 4.91; N, 16.50%.

### Biological assay

#### Animals

To define the minimum total sample size and the average number for each group the *a priori* test with the following parameters was conducted: anticipated effect size (Cohen's *d*): 0.7; desired statistical power level: 0.8; probability level: 0.05. The *a priori* test calculation returned the following values: minimum total sample size (one-tailed hypothesis)^38^; minimum sample size per group (one-tailed hypothesis)^39^.

According to *a priori* test results, experiments were conducted on young adult male Sprague–Dawley rats (5 rats per group), weighing 140–170 g. The animals were housed at cages in a temperature controlled (25 ± 1 °C) environment and were fed *ad libitum* with standard laboratory chow and allowed free access to water. This investigation conforms to the ethical Guide for the Care and Use of Laboratory Animals published by the US National Institutes of Health (NIH Publication No. 85–23, revised in 1996). The animal protocol is in accordance with the Animal Ethical Care regulations in the Faculty of Pharmacy, Helwan University.

#### Assessment of anti-inflammatory activity

Compounds (equimolar to the reference drug) were dissolved in DMSO and administrated subcutaneously. One hour later, paw oedema was induced by subplantar injection of 0.1 ml of 1% carrageenan (Sigma-Aldrich, St. Louis, MO) into the right hind paw. Paw volume was measured using a water plethysmometer (Basile, Comerio, Italy). The difference between the right and left paw volume was measured at 1, 2, 3, and 4 h after induction of inflammation.

The control group (five rats per group) received DMSO subcutaneously and carrageenan in the subplantar region. Results were expressed as percentage inhibition of inflammation. Ibuprofen (70 mg kg^−1^) was used as the reference drug^40^.

#### Biochemical assay

Drugs capacities to inhibit COX-1, COX-2, and 5-LOX enzymes were assessed using ELISA kits; COX-1 (human) Inhibitor Screening Assay Kit (Item № 701070), COX-2 (human) Inhibitor Screening Assay Kit (Item № 701080), and 5-LOX Inhibitor Screening Assay Kit (Item № 760700) from Cayman (Ann Arbor, MI). The used protocol was according to the manufacturer protocol guide and instructions using ELISA plate reader.

##### Statistics

All assays results are expressed as mean ± standard error of the mean (SEM). The comparisons between the control and the treatment groups were carried out using One-way ANOVA using a statistical package (SPSS version 17.0). A *p* value of <0.05 was considered significant.

### Molecular modelling

All molecular modelling work was performed using SYBYL-X package (www.certara.com) installed with licence to the Faculty of Pharmacy, King Abdulaziz University on Windows 7-operated computer, equipped with Samsung SyncMaster 2233RZ 120 Hz LCD Display™ (3D ready) and Nvidia Geforce 3D Vision Glasses Kit™.

#### Preparation of protein and ligands

Crystal structures were downloaded as .pdb files from Protein Databank Website (www.rcsb.org). The initial biopolymers were simplified by deleting all but one monomer in the quaternary structures and then prepared for docking. The previous two steps were performed using “Biopolymer” Tools.

The Ligand Structures Library was built on Chemsketch^41^ and saved as .sdf files. The structures were converted to 3D and optimised using SYBYL-X’s Concord embedded in “Prepare Ligands” tools.

#### Surflex docking

Docking was performed using Surflex programme embedded in “Dock Ligand” tools. First, the target previously prepared protein was selected and underwent final preparation for docking. Surflex docking was performed after protomol generation on ligand mode.

#### Manual docking

##### Ligand preparation

The manual docking was used in case no ligand was present to guide Surflex automatic docking procedure. This protocol was used only for docking experiments of syn-**7a**, anti-**7a**, and **8c** to the active site of 5-LOX crystal structure (PDB Code 3O8Y) because it contains no ligand to guide a Surflex automatic docking. In this regards, ligands prepared above we further optimised to the global minimum conformation by energy minimisation tools until the global minimum is reached (Termination: Gradient).

##### Docking

We used three-step, visually-guided procedure (Place-Merge-MD) as follows.

*1. Place*. Prepared protein structure (as described above) and the intended ligand were imported to two different SYBYL-X screen levels. The ligand was moved until it was visually entered the active site. Careful inspection of features in the ligand and residues around to ensure maximum positive interactions with the least clashes with the active site residues.

*2. Merge.* When convinced that the ligand is situated in the best docking position, the ligand was merged into the protein screen. To confirm that the ligand has no serious clash with the active site residues, AAs with 5 Å sphere distance around the docked ligand were unhidden. If a clash existed, another round of moving the ligand was performed and the procedure is repeated until the least possible clash is reached. After merge, the energy of the complex it is measured and compared to uncomplexed protein. The process is repeated several times with different docking modes. The best docking complexes pose according to visual inspection of clashes and energy computation were retained and compared. The poses ranked according to their energies (first priority) and positive interactions of the ligand with the active site.

*3. Molecular dynamics (MD)*. Molecular dynamics was performed to check the stability of the complex generated by manual docking according to the following protocol with time length 1000 fs at 300 K and 0 atm pressure. Snapshots every 5 fs were taken and inspected visually. The best snapshot in energy is considered provided that a minimum movement of the protein loops are observed.

## Results

### Chemical synthesis

**Scheme 1.**
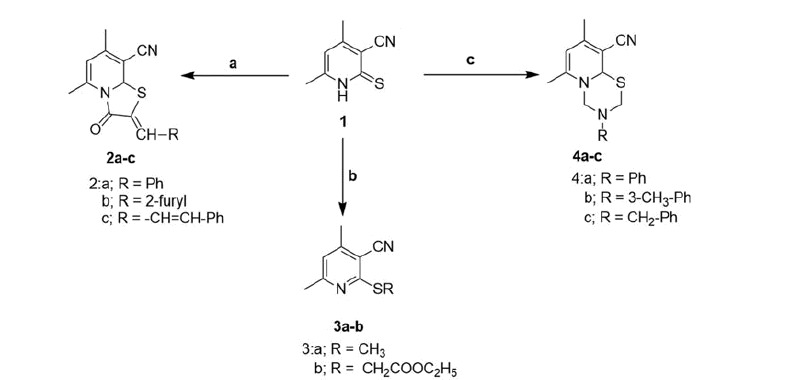
Utilisation of compound **1** to produce series **2**–**4** (a = ClCH_2_COOH, Ac_2_O, AcOH, NaOAc along with an aldehyde RCHO, b = RX where X = Br or I, c = RNH_2_ and HCHO).

Thienopyridine synthesis has been previously reported^42^. Synthesis of target anti-inflammatory compounds was accomplished *via* the key intermediate pyridin-2-thione (compound **1**), which was prepared according to the reported method^37^. Compound **1** was then utilised in different reactions to yield three different series of compounds^2–4^. Condensation of **1** with chloroacetic acid and certain aldehydes in a mixture of acetic acid and acetic anhydride gave the thiazolopyridine derivatives **2a**–**c**^43^. Compound **1** was also treated with different alkylating agents to afford the corresponding 2-alkylthio analogues **3a**–**b**. Condensation of **1** with formalin or certain primary amines afforded the pyridothiadiazine derivatives **4a**–**c** (Scheme 1).

**Scheme 2.**
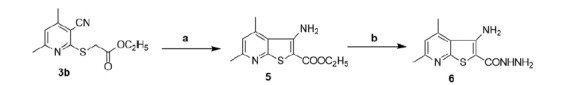
Conversion of **3b** to thienopyridine derivatives (a = NaOEt/EtOH, b = NH_2_NH_2_).

Compound **3b** was subsequently converted to the thienopyridine derivatives **5** via reaction with NaOEt/EtOH. Compound **5** was treated with NH_2_NH_2_ to yield the corresponding hydrazide **6** (Scheme 2)^37^.

**Scheme 3.**
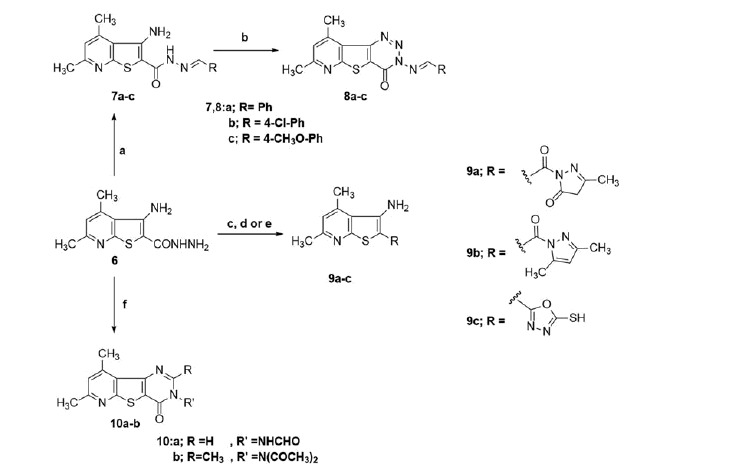
Various reactions of compound **6** to afford the target compound series **7**–**10** (a = RCHO, b = NaNO_2_, HCl, c = ethyl acetoacetate, d = acetylacetone, e = CS_2_, f = HCOOH or Ac_2_O).

The intermediate compound **6** was used as a diversity precursor to prepare a number of target compounds’ series^7–11^. Compound **6** was reacted with certain aldehydes to give the corresponding arylidene hydrazide **7a**–**c** which were subsequently reacted with NaNO_2_/HCl to afford the pyrido[3′,2′:4,5]thieno[3,2-d][1,2,3]triazin-4(3*H*)-one **8a**–**c**^44^. In addition, condensation of compound **6**, with ethyl acetoacetate, acetylacetone or carbon disulphide to afford the derivatives **9a**–**c** respectively. Finally, the reaction of **6**, separately with HCOOH and acetic anhydride afforded the pyridothienodiazine analogues **10a** and **10b**, respectively (Scheme 3).

### Biological screening

#### *In vivo* anti-inflammatory assay

**Figure 3.**
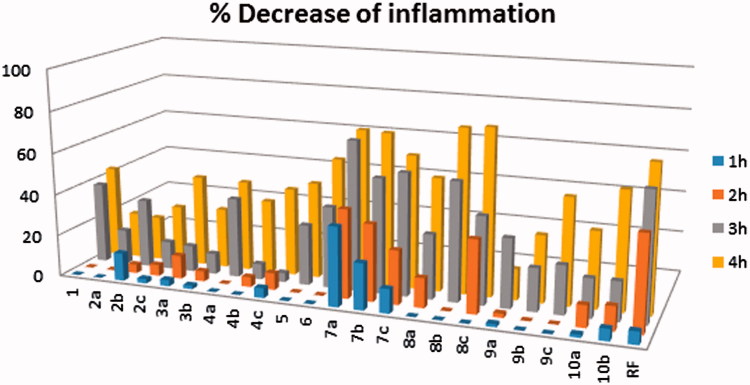
*In vivo* anti-inflammatory assay of thieno[2,3-*b*]pyridine derivatives. The anti-inflammatory potency is proportional to the height of the bar (inhibition %). The height of the bars (inhibition%) = (swell.drug/swell.control)100. Swell = mean difference in rat paw volume between right and left paw. RF = ibuprofen (reference drug). For more details, refer Supplementary Table S1.

The obtained compounds were tested for their anti-inflammatory activity using the carrageenan-induced rat paw oedema assay. Compound potency and duration of action were compared with those of the reference compound ibuprofen. The *in vivo* anti-inflammatory activity is shown in Figure 3.

Compounds **7a**, **7b**, **8b**, and **8c** induced a satisfactory anti-inflammatory activity, exceeding that of ibuprofen at the 4 h time interval. The compounds produced 73.5, 73.2, 78.2, and 79.4% inhibition respectively, compared to 69.5% inhibition for ibuprofen. The time–activity profiles of these compounds are commensurate to that of ibuprofen, varying only in the onset of action. Compounds of series **7** showed a more rapid onset than ibuprofen, while those of series **8** showed a much slower onset (Supplementary Table S1).

#### *In vitro* inhibition assays of 5LOX, COX-2, COX-1 enzymes

The compounds belonging to series **7** and **8** were subjected to enzyme assay investigations against 5-LOX, COX-1, and COX-2. Those compounds were chosen based on the demonstrated profound anti-inflammatory activity in the above *in vivo* test.

## Discussion

The phenotypic screening preceding isolated enzyme assays provided an opportunity to shift focus towards series of compounds that exhibited higher potential therapeutic benefit. The structure–activity relationship (SAR) of screened compounds for *in vivo* anti-inflammatory activities favoured thieno[2,3-*b*]pyridines over other scaffolds such as **2** and **4**. The 5-LOX inhibition assay, however, favoured the hydrazides **7** as the most potent compound, **7a**, is 35 and 87 times higher than the tricyclic derivatives **8a** and **8c**, respectively. A similar result was noticed in the COX-2 inhibition assay, albeit to a lower extent. In this regard, **7a** inhibited COX-2 at 3.6 and 1.8 times higher than **8a** and **8c**, respectively.

Results, displayed in Table 1, were interesting since potency level, as well as selectivity between the two series **7** and **8**, explained the potential of these compounds. The *in vitro* enzyme inhibition assays revealed that compounds **7a** and **7b** realised higher potency and selectivity towards 5-LOX and COX-2 over COX-1. They are also higher than the compounds **8b** and **8c** as well as ibuprofen^45^ in their 5-LOX and COX-2 inhibition potency. Compounds **7a** and **7b** are also more selective than the other three compounds towards COX-2 than COX-1 with selectivity index COX-2/COX-1 = 4.6 and 4.2 for **7a** and **7b**, respectively. Compound **8b** and **8c** demonstrated approximately 2-fold more affinity towards COX-1 over COX-2.

**Table 1.** Enzyme inhibition assay results for COX-1, COX-2, and 5-LOX along with per cent inflammation reduction at the 4 h time interval in the rat paw oedema assay.

Compound	5-LOX IC_50_ (µM)	COX-2 IC_50_ (µM)	COX-1 IC_50_ (µM)	inflammation reduction % (4h)
**7a**	0.154	9.988	45.869	73.54
**7b**	0.279	7.524	31.797	73.02
**8b**	13.520	35.990	19.902	78.23
**8c**	5.419	18.992	7.663	79.36
**Ibuprofen**	0.436	43.628	31.945	69.52

All standard error of means (SEM) are lower than 10%.

The lack of proportionality between *in vitro* and *in vivo* readouts can be attributed to several common factors, mainly pharmacokinetic parameters (absorption, distribution, metabolism, and excretion) that account for discriminations in drug efficacies^46^.

A structure-based analysis by docking selected compounds in the active site of 5-LOX, COX-2, and COX-1 was conducted to propose an understanding of the enzyme inhibition assay results. Crystal structures from the Protein Databank website (www.rcsb.org) were downloaded and employed in the study, accession codes: 3O8Y for 5-LOX^47^, 4PH9 for COX-2^48^ and 1EQG for COX-1^38^. All the molecular modelling work was carried out using SYBLYL-X v.2.1 package licenced to King Abdulaziz University according to methodologies and protocols described in the “Experimental” section and detailed in Supplementary Material.

Among all crystal structures of 5-LOX, only three entries belong to *H. sapiens* 5-LOX (Entries: 3O8Y, 3V98, and 3V99). All of these crystal structures are mutated by replacing three lysines (K^653^KK^635^) into Glutamic acid–Aspargine–Leucine (ENL). The latter two entries had additional mutation S663D which resulted in the loss of 5-LOX activity (converted to 15-LOX-like activity)^49^. Therefore, we settled with 3O8Y as our target for docking experiments.

The active site of 5-LOX is framed by two α-helices, the α2-helix and the arched helix, forming an elongated U-shaped hydrophobic cavity that accommodates the substrate AA, a 20-carbon polyunsaturated fatty acid^50^. There is a catalytic iron nearby the active site but access to this iron is regulated by the side chains of Phe-177 and Tyr-181 (the FY cork), which also caps the cavity once the substrate enters the active site^47^. The defined conformation of this active site helped in understanding the clearly higher 5-LOX inhibitory activities of **7a** and **7b** (IC_50_ are at nanomolar level) over the cyclised analogues **8b** and **8c** (IC_50_ are at micromolar level).

To rationalise the higher activity of **7a** and **7b** against 5-LOX, a study of different **7a** conformers was carried out. The *anti*-conformer is the calculated global minimum and it was found to be 3.8 kcal/mol less energy than the *syn-*conformer. This *anti*-conformer can make an intramolecular hydrogen bonding between the 3-amino and the carbonyl oxygen, more efficiently than that of the *syn*-conformer (Supplementary Material). It is therefore proposed that the *anti*-conformer of **7a** attains a higher population.

**Figure 4.**
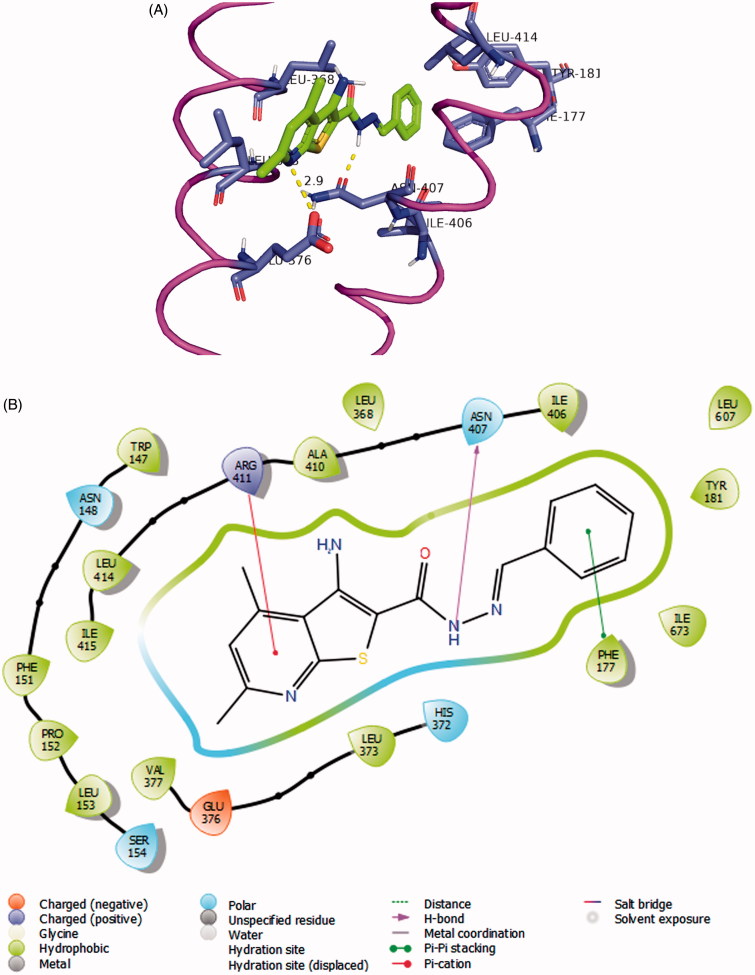
(A) Compound **7a** (coloured green) docked into the active site of 5-LOX. The α2 helix is seen to the left and the arched helix is located to the right (both coloured magenta). The hydrophobic residues interacting with the ligand Leu-368, Leu-373 (of α2 helix), Ile-406, and Leu-414 (of arched helix) are shown. The FY cork residues Phe-177 and Tyr-181 are seen to the top of the image. The graphics of this image was generated using Pymol free software (https://pymol.org/2/). (B) 2D representation of the interactions of **7a** with the active site of 5-LOX generated using Maestro visualiser.

Interestingly, the pyrido[3',2':4,5]thieno[3,2-d][1,2,3]triazin-4(3H)-one derivatives^8^ attain a global minimum conformation that well overlaps with the *syn-*conformation of **7a**. For docking experiments into 5-LOX crystal structure, we used visually guided manual docking followed by molecular dynamics (MD), since the active site contained no ligand to guide the automatic docking process by Surflex^51^. All three conformers (*anti*-**7a**, *syn-***7a** and **8c**) could rest inside the active site of the human 5-LOX in a hydrophobic tunnel between the α-2 helix and the arched helix considering the least steric clashes with surrounding residues (Figure 4). Moreover, according to the proposed model, our compounds established several hydrophobic contacts within convenient van der Waals distances with hydrophobic residues lining the active site. The *anti*-conformer of the most active compound **7a** made attractions with side chains of Leu-373, Leu-368, Ile-406, Leu-414, and Leu-607. In addition, *anti-***7a** is theorised to form a crucial hydrogen bonding interaction through the hydrazide NH to Asn-407, an important residue of the arched helix that contributes to the shape of the active site. Surprisingly, the pyridine nitrogen of within the thieno[2,3-*b*]pyridine nucleus formed what is possibly a very important ionic interaction with Glu-376 side chain carboxylate with all three docked structures (*anti-*, *syn-***7a** as well as **8a**).

Compound **8b** was approximately 90 times less potent than **7a** in inhibiting 5-LOX in enzyme inhibition assays. This can be explained using the structure-based analysis of an affinity model with 5-LOX active site. As mentioned above, both **8b** and **8c** resemble the *syn*-conformation of **7a**. These entities were generally able to maintain the major polar and non-polar interactions with the active site residues. However, **8b** approached Ile-415 and Leu-368 side chains inconveniently, thus making possible clashes with the active site in this docking mode (Supplementary Material). To be accommodated in the active site, significant shifts in the primary backbone protein loops have to be made after the MD step. Therefore, it should be challenging to predict the actual binding mode of this compound in this less flexible closed active site.

According to this model, it is noted that the 4,6-dimethylthieno[2,3-*b*]pyridine scaffold has a good physicochemical balance in the molecular properties as it rests in a dominantly lipophilic pocket. The two methyl groups bolstered the aromatic ring hydrophobic attractions with Leu-368 and Leu-373 side chains (residues of α2 helix, Figure 4) while the pyridine nucleus conferred a much needed polar character to our compounds.

**Figure 5.**
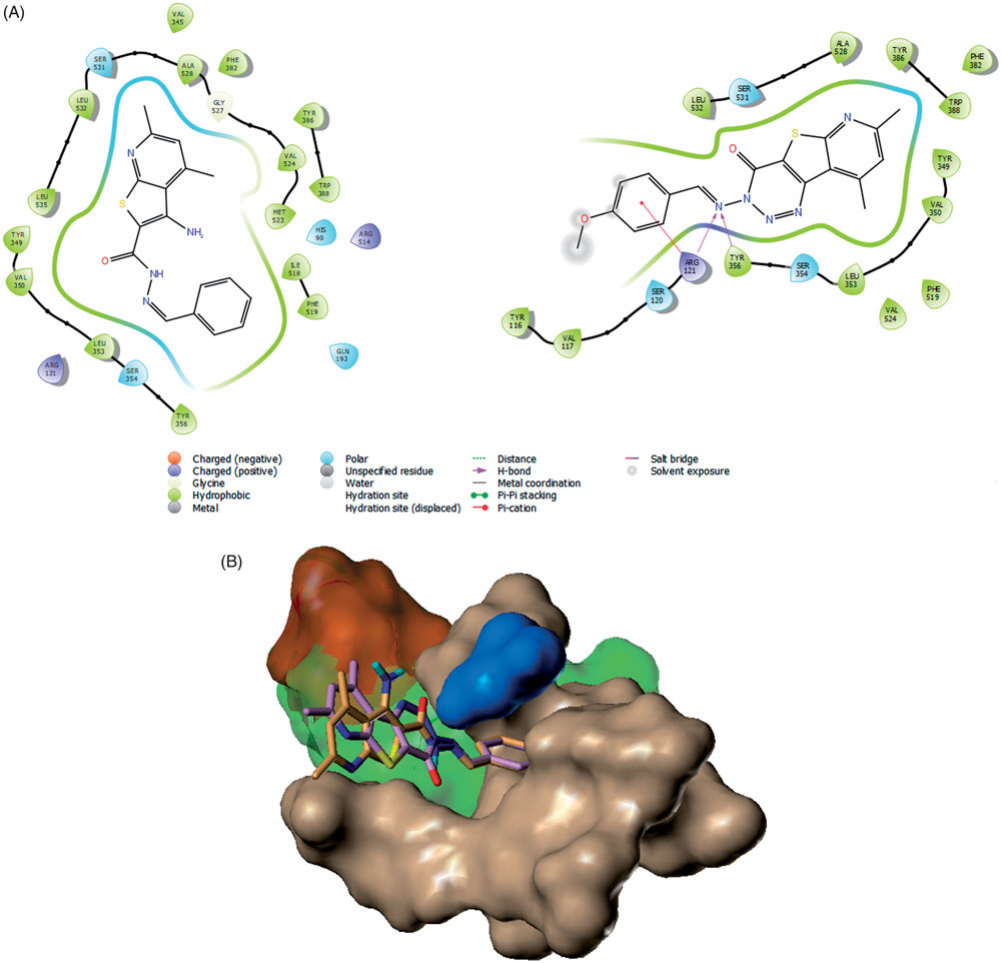
Docking of **7a** and **8b** to COX-2 crystal structures. (A) 2D representation of the binding modes of compounds **7a** (left) and compound **8c** (right) with the active site of COX-2 generated using Maestro visualiser. (B) Model of docking of **7a** (violet) and **8c** (Orange) on COX-2 crystal structure (Code: 3OLU). The active site pocket is coloured green as determined by Surflex’s protomol except the side pocket, which was given an orange surface. Surface of the active site’s main hydrophobic channel residues are coloured coffee-brown. Ser-530 is given a blue surface.

Lead compounds’ affinity towards COX-2 were investigated in a virtual screening model using Surflex docking application embedded in SYBYL-X package and the Surflex docking scores were compared^51^. For this purpose, we initially chose a crystal structure of COX-2 containing ibuprofen (Code: 4PH9), the reference drug for both *in vivo* and *in vitro* screenings. Our analysis suggests possible binding modes of the tricyclic analogue **8c** reasonably. The active site of COX-2 is composed of four main regions according to the crystal structures; the main hydrophobic channel, the polar mouth area (mainly side chains of Arg-120 and Tyr-355), the selectivity side pocket and another smaller hydrophobic pocket near the mouth area^52^. Therefore, in a previous model, we reported that a pyridylurea derivative of naproxen binds possibly by inserting the naphthalene moiety properly deep in the main channel. Meanwhile, the urea group makes several hydrogen bonding with the polar area near the active site mouth^28^. In our present work, compound **8c** was predicted to insert the bulky thieno[2,3-*b*]pyridine moiety into the hydrophobic channel and make hydrophobic contacts with side chains of Val-344, Tyr-348, Val-349, Phe-385, Trp-387, Leu-531, and Leu-534 (Figure 5). The triazine ring and imine group form a network of hydrogen bonding with the guanidine side chains of Arg-120 and the phenol group of Tyr-355 (similar to ibuprofen’s carboxylate interactions). The terminal aryl group of Schiff base was directed to the outside of the protein surface possibly because this tricyclic ligand does not enjoy much flexibility.

A limitation of this model is that it was not successful in describing the good binding of **7a**, from our perspective. The top-scoring pose (Table 2) of this bicyclic compound, being more flexible than **8c**, was placed by Surflex as an *syn*-conformer while directing its thieno[2,3-*b*]pyridine moiety towards the hydrophobic channel. It also forced the terminal phenyl group of the Schiff base to an *eclipsed* torsion that penetrated the characteristic side pocket of COX-2. We had some concerns about this suggested model of **7a** because it was predicted to be half the potency of **8c** according to Surflex scores (Table 2). In addition, the docking conformation, though filled the side pocket, did not make any significant polar interactions (*cf.***8c**) with the active site polar region. This limitation highlights that this model is merely a speculative trial to explain the enzyme inhibition activities of the reported compounds.

**Table 2.** Surflex total scores of docked compounds into COX-2 (Code 4PH9 and 3OLU) and COX-1 (1EQG).

Compound	Surflex docking score		
	4PH9	3OLU	1EQG
**Ibu**	7.5	6.8	6.5
**8d**	7.7	7.2	4.6
**8b**	5.7	6.3	3.9
**7a**	4.0	5.7	1.3
**7b**	2.3	6.2	1.2

Surflex docking score = Calculated –log *K*_d_

**Figure 6.**
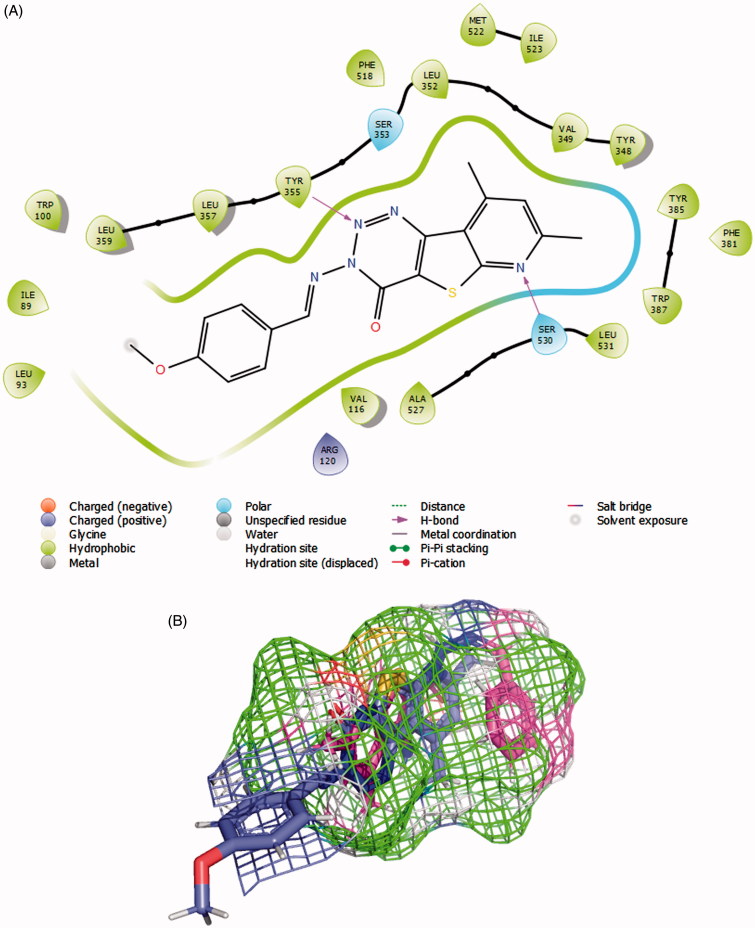
(A) **8c** (violet) and **7a** (magenta) in COX-1 active site as determined by Surflex Graphics of this image was generated using Pymol free software (https://pymol.org/2/). (B) 2D diagram showing the putative model of binding mode of compound **8c** with COX-1 active site (PDB Code: 1EQG).

We suspected that the protomol formation did not help the model prediction efficiency because the process is known to be affected by the co-crystallised ligand’s size and shape^51^. In addition, the main channel of the active site of this crystal structure is seemingly shrunk tightly around the small isobutyl tail of ibuprofen, the co-crystallised ligand. Therefore, we decided to perform the modelling work on another COX-2 crystal structure (PDB Code: 3OLU) that contained arachidonic acid glyceryl ester as a co-crystallised ligand of larger size^53^. The larger ligand gave rise to a significantly longer but narrower protomol in the main channel pocket (Supplementary Material). The docking experiments of the four compounds to the active site returned different predictions of binding modes (Figure 6(B)). The Schiff base group was inserted inside the main hydrophobic channel (reverse mode to the previous model on 4PH9). Both ligands (**7a** and **8c**) made hydrogen bonding with Ser-530 side chain. It is worthy to mention that Ser-530 is a residue involved in the catalytic peroxidation of AA substrate, forming a hydrogen bond with the carboxylic group of diclofenac, and being acetylated by acetylsalicylic acid as proven by structural biology reports^54^. Compound **7a** in this model existed in the previously predicted more probable *anti*-conformation (∼17 kcal/mol lower than that of the first docking model). The bulkier thieno[2,3-*b*]pyridine moiety was oriented towards the outside of the active site with shallow penetration of the selectivity pocket. Based on these assumptions, the model also helped explain the slight selectivity of **7a** to COX-2 over COX-1 (Will be discussed later in further details). In our analysis, this model had improved predictions but it is not free from concerns. The much lipophilic dimethyl pyridyl ends are projecting outside the active site to the aqueous medium. In addition, the impact of the substituents on the Schiff base phenyl groups on the experimental activities are not supported by this model. Finally, the calculated scores in this model still rank **8b** and **8c** higher than **7a** and **7b** but the differences in the total Surflex scores are not as drastic as the first model (for the 4PH9 structure) (Table 2). Since the experimental differences between the ligands are not drastic either, the model might be considered qualitatively aligned with the experimental results to provide a possible explanation of the relative compounds’ activities. We expect that the scoring, as well as the binding modes, may vary if we use different crystal structure entries among the many available in the PDB website.

Modelling the COX-1 inhibition was performed on a crystal structure that also contained ibuprofen as a ligand (PDB Code: 1EQG)^38^. It is established that there are several common features between COX-1 and COX-2 active sites, the main hydrophobic channel, the catalytic Ser-530 and the mouth having polar residues such as Arg-120. However, COX-1 is characterised by the absence of the side pocket and by a narrower main hydrophobic channel^55^. In docking experiments, Surflex successfully granted **8c** the highest scoring on COX-1, which aligns with experimental results (Tables 1 and 2). The dimethyl pyridine moiety reached the hydrophobic bottom of the main channel and was adjusted to form a hydrogen bond with Ser-530. The terminal triazine ring formed a bifurcate hydrogen bonding with Tyr-355. The rigid nature of **8c** added convincing evidence for a high probability of its postulated binding mode. Meanwhile, Surflex placed the flexible Schiff base end of **7a** to the bottom of the active site and twisted the imine side chain to fill this pocket. This way, the *anti*-conformer of the ligand was totally immersed inside the active site assuring that the dimethylpyridyl end is exposed to a hydrophobic microenvironment. No effective polar contacts were observed for the ligand (Figure 6). We checked if the linear global energy minimum would give a better binding quality by a manual docking experiment but found serious steric clash with Val-518 (Supplementary Material).

In conclusion, this modelling work provided a hypothetical rationale of speculative binding modes of the title compounds and improved our understanding of the experimental results because it reasonably aligned these calculations with experimental results within a plausible margin of deviation.

## Supplementary Material

IENZ_1457657_Supplementary_Material.pdf
