# Supplementary material for: Molecular modelling insights into a physiologically favourable approach to eicosanoid biosynthesis inhibition through novel thieno[2,3-b]pyridine derivatives
Source: J Enzyme Inhib Med Chem. 2018 Apr 13;33(1):755–67. doi: 10.1080/14756366.2018.1457657 (PMC6009894; doi:10.1080/14756366.2018.1457657)
Supplement: IENZ_1457657_Supplementary_Material.pdf [file IENZ_A_1457657_SM2329.pdf]

Supporting Information

Molecular Modelling Insights into a Physiologically Favourable Approach to Eicosanoid Biosynthesis Inhibition through Novel thieno[2,3-*b*]pyridine Derivatives

Mosaad S. Mohamed[1], Yara E. E. Awad[1], Hatem K. Amin[2] and Moustafa E. El-Araby*[1]

[1]*Department of Pharmaceutical Organic Chemistry, Faculty of Pharmacy, Helwan University, Cairo, Egypt*

[2]*Department of Biochemistry and Molecular Biology, Faculty of Pharmacy, Helwan University, Cairo, Egypt*

1. In vivo anti-inflammatory screening

Table SI-1

	**Inhibition of acute inflammation♦ %**							
	**1 h**		**2 h**		**3 h**		**4h**	
	**Swel** ±SE	**% inh**	**Swel** ±SE	**% inh**	**Swel** ±SE	**% inh**	**Swel** ±SE	**% inh**
**1**	**0.503** ± 0.040	**0**	**0.353** ±0.052	**0**	**0.33** ±0.033	38.725	**0.353** ±0.018 a***	43.91
**2a**	**0.457** ± 0.088	**0**	**0.595** ±0.036	**0**	**0.453** ±0.024 b*	16.605	**0.491** ±0.014 b***	21.95
**2e**	0.199 ±0.022	13.4	0.249 ±0.041	4.23	0.365 ±0.033	32.84	0.497 ±0.002 b***	21.06
**2f**	0.223 ±0.022	2.16	0.245 ±0.029	5.37	0.471 ±0.065	13.22	0.458 ±0.093	27.65
**3a**	**0.382** ±0.049	**0**	**0.363** ±	**0**	**0.476** ±0.038 b*	12.438	**0.354** ±0.028 a***	43.7
**3c**	0.2255 ±0.02	1.73	**0.2407** ±0.024	**4.92**	0.487 ±0.092	9.9	0.432 ±0.019	28.67
**4a**	**0.394**	**0**	**0.407** ±0.23	**0**	**0.336** ±0.036	38.174	**0.356** ±0.048	43.38

Supporting Information

	± 0.015						a^{***}	
**4c**	0.45 ±0.076	**0**	0.22 ±0.063	4.78	0.213 ±0.055	7.39	0.409 ±0.013	35.08
**4f**	**0.5** ± 0.063	**4.78**	**0.239** ±0.083	**8.08**	**0.52** ±0.038	4.35	**0.365** ±0.031 a^{***}	42.06
**5**	**0.393** ±0.056	**0**	**0.46** ±0.023	**0**	**0.386** ±0.04	28.921	**0.34** ±0.045 a^{***}	46.03
**6**	**0.410** ±0.032	**0**	**0.486** ±0.02	**0**	**0.332** ±0.029	38.84	**0.26** ±0.020 a^{***}	58.67
**7a**	**0.143** ± 0.059	37.68	**0.15** ±0.042	42.3	**0.156** ±0.052 a^{***}	71.2	**0.16** ±0.040 a^{***}	73.54
**7b**	0.17 ± 0.035	22.1	0.16 ± 0.039	35.62	0.25 ± 0.018	54.75	0.17 ± 0.072 a***	73.02
**7d**	**0.203** ± 0.025	11.59	**0.193** ±0.064	25.64	**0.226** ±0.018 a^{***}	58.3	**0.23** ±0.036 a^{***}	63.49
**8a**	**0.256** ± 0.020	**0**	**0.223** ±0.053	14.1	**0.376** ±0.052	30.75	**0.29** ±0.051 a^{***}	53.96
**8b**	0.23 ± 0.026	0	0.27 ± 0.003	0	0.24 ± 0.017	56.54	0.14 ± 0.046 a***	78.23
**8d**	**0.246** ± 0.058	**0**	**0.17** ±0.042	34.61	**0.316** ±0.09	41.78	**0.13** ±0.013 a^{***}	79.36
**9**	0.227 ±0.018	1.45	0.255 ±0.062	1.92	0.374 ±0.027	32.84	0.537 ±0.042 b^{***}	14.76
**10**	**0.476** ± 0.051	**0**	**0.498** ±0.72	**0**	**0.433** ±0.031	20.4	**0.428** ±0.023 a^{*},b^{***}	32.01
**11a**	0.227 ±0.021	1.45	0.233 ±0.008	10.38	0.44 ±0.013	18.5	0.4 ±0.041	36.5
**11b**	**0.217** ± 0.022	**5.65**	**0.23** ±0.087	**11.45**	**0.443** ±0.100	18.56	**0.275** ±0.071 a^{***}	56.29
**13**	**0.506** ±0.076	**0**	**0.583** ±0.051	**0**	**0.418** ±0.027	23.1	**0.31** ±0.006 a^{***}	50.79
Ibuprophen	0.216	6.08	0.14	45	0.214	60.66	0.192	69.52

Supporting Information

	± 0.032		±0.55		±0.019		±0.015	
**control**	**0.23** ± 0.003		**0.26** ±0.037	**0**	**0.544** ±0.08		**0.63** ±0.03	

- **♦ solvent: 2.5 mL DMSO. Dose: 70 mg kg-1 ibuprofen and the equivalent amount of**
- **tested compounds.**
- **Swel= mean difference in rat paw volume between right and left paw.**
- **Control (DMSO) is negative (has no anti-inflammatory activity).**
- **%inhibition =(1-rt/rc)x100 [rt= mean of tested group; rc= mean of control group]**
- **a: Significantly different from control, b: Significantly different from ibuprofen**
- **as indicated: *P < 0.05; ** P<0.01; ***P < 0.001**
- **Swel = swelling, SE = Standard Error, %inh = % inhibition**

2. Molecular Modeling

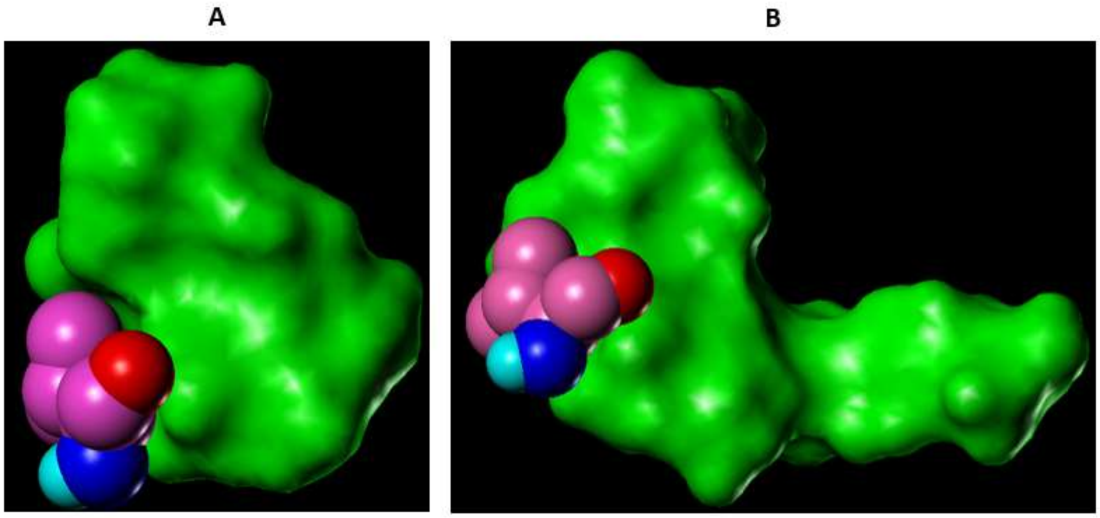

Figure SI-1. The active site shape as identified by Surflex for COX-2 two different crystal structures: (A) Pdb Code: 4PH9 and, (B) PDB Code: 3OLU. The residue shown in spheres is the side pocket gate keeper Val-523.

Supporting Information

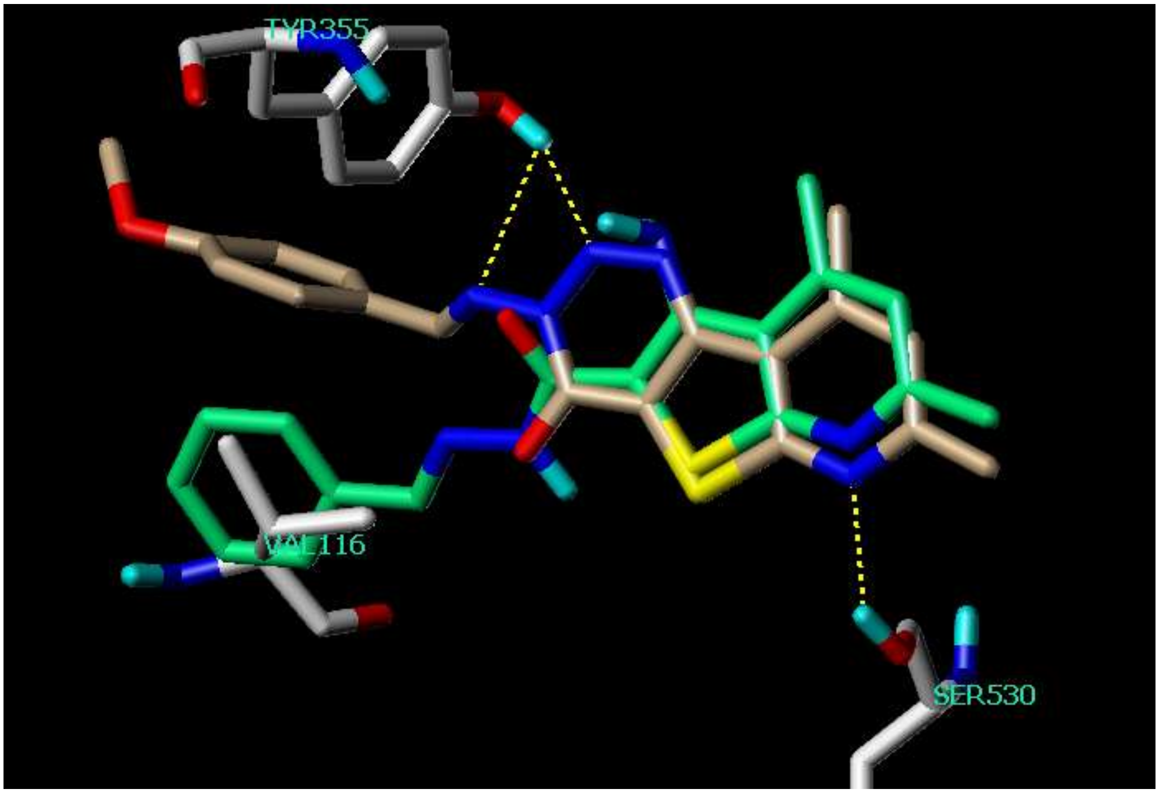

Figure SI-2. The great clashes between the phenyl group of 7a (global minimum *anti* conformer, carbon are colored green) and the active site of COX-1 when the thieno[2,3-b]pyridine nucleus are superposed. Compound 8d as predicted by Surflex docking is shown in golden color and it is safe.

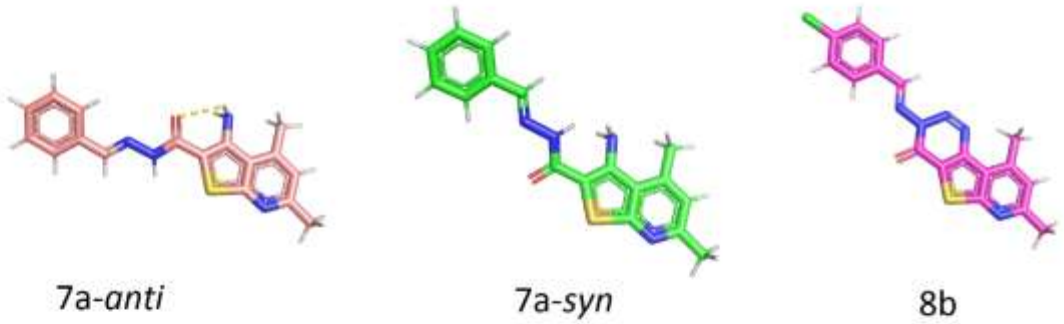

Figure SI-3. Possible conformers of compound 7a. The dashed line in the left (*anti*) conformers denote hydrogen bonding.

3. Details of Methods and Protocols of Molecular Modelling

A. Hardware: Common Desktop run on Window 7 operating system and equipped with Samsung SyncMaster 2233RZ 120 Hz LCD Display™ (3D ready) and Nvidia Geforce 3D Vision Glasses Kit™.

Supporting Information

B. Software: SYBYL-X package installed with license to the Faculty of Pharmacy, King Abdulaziz University (40 license numbers are available).

C. Protocols.

Preparation of Proteins

Crystal structures were downloaded as .pdb files from Protein Databank Website (www.rcsb.org). The initial biopolymers were simplified by deleting all but one monomer in the 4ry structures using “Biopolymer” tools (Composition>Delete Monomers). The protein was prepared using “Biopolymer” tools (Biopolymer>Prepare Structure>Structure Preparation Tools). The following processes were used in preparation:
Analyze Selected Structure

Termini Treatment: Charged (N Terminal is given +ve and C Terminal is –ve charge)
Add Hydrogens: All, Hydrogen Orientation in Waters: H-Boniding
Set protonation Type: His (H on N^{τ})
Add Charges: Biopolymer: MMFF94 and Ligands: Gasteiger-Marsili
Fix Sidchain Amides: Asn and Gln
Fix Sidechain Bumps
Staged Minimization: Performed
Minimization Details
Method: Powell
Initial Optimization: None
Termination: Gradient 0.05 kcal/(mol*A)
Max Iterations: 100
Non-Bonded Reset: 10
Max Displacement: 0.01
Min Energy Change: 0.05
LS Accuracy: 0.001
LS Step Size: 0.001
Status Update: 1
Graphics Update: 1
Non-Bonded Reset: 10
RMS Displacement: 0.001
Force Field: Tripos
Charges: Use Current
Dielectric Function: Constant = 1.00
Non-Bonding Cutoff: 8.0 A
Other less important parameters: SYBYL-X default
Stage Orders:
1. Minimize Biopolymer Hydrogens
2. Minimize Waters
3. Minimize Sidechains
4. Minimize Biopolymer with C_{α}

5. Minimize Ligand
6. Minimize All

Preparation of Ligands

The Ligand Structures Library were built on Chemsketch [ref] and saved as .sdf files. The structures were opened by SYBYL's "Prepare Ligands" tools and converted to a minimized 3-dimensional structures using Concord.
Docking was performed using Surflex program embedded in "Dock Ligand" tools. First, the target previously prepared protein was selected and underwent final preparation for docking:
Delete Waters: Near Active Site
Delete Ligands: All except the ligand present in the intended pocket
Select Ligand: Ligand selected in intended pocket

Surflex Docking
Docking Mode: Surflex (SFXC)
Protomol Generation Mode: Ligand
Other less important parameters are default

Manual Docking
The manual docking was used in case no ligand was present to guide Surflex automatic docking procedure. This protocol was used only for docking experiments of syn-7a, anti-7a and 8c to the active site of 5-LOX crystal structure (PDB Code 3O8Y) because it did not contain a ligand to guide a Surflex automatic docking.

Preparation of Ligands for manual docking

Ligands prepared above were further optimized to the global minimum conformation by energy minimization tools as follows:

Method: Powell
Initial Optimization: None
Termination: Gradient 0.01 kcal/(mol*A)
Max Iterations: till gradient
Non-Bonded Reset: 10
Max Displacement: 0.01
Min Energy Change: 0.05
LS Accuracy: 0.001
LS Step Size: 0.001
Status Update: 1
Graphics Update: 1
Non-Bonded Reset: 10
RMS Displacement: 0.001
Force Field: Tripos
Charges: Gasteiger-Marsili
Dielectric Function: Constant = 1.00

Supporting Information

Non-Bonding Cutoff: 8.0 A
Other less important parameters: SYBYL-X default

Docking

We used three-step, visually-guided procedure (Place-Merge-Measure) as flows:

1. *Place* ligand into the active site.
Prepared protein structure (as described above) was imported to a SYBYL-X screen level (M1 for example). The crystal structure ligand was extracted to a different screen level (M3 for example). To make visualization more efficient, relevant active site residues that might impact the docking and possibly interact with a ligand were selected and kept on the screen (Residues Number 177, 181, 364-378, 402 to 414, 432, 599-607 and the catalytic iron), according to the described by Gilbert et al [Science. 2011;331(6014):217-9]. Other AAs in the sequence were hidden (but not deleted). An optimized ligand was imported to a different screen level (M2 for example). The ligand was moved until it was visually entered the active site. Careful inspection of features in the ligand and residues around to ensure maximum positive interactions with the least clashes with the active site residues. When convinced that the ligand is situated in the best docking position, the ligand was merged into the protein screen using Merge Tools (Edit>Merge). To confirm that the ligand has no serious clash with the active site residues, AAs with 5 Å sphere distance around the docked ligand were unhidden. If a clash existed, another round of moving the ligand was performed and the procedure is repeated until the least possible clash is reached. After merge, the energy of the complex it is measure according to the following protocol:

Compute>Energy

Force Field: Tripos
Charges: Use Current
Dielectric Function: Constant = 1.00
Non-Bonding Cutoff: 8.0 A
The complex (after merge) energy was compare to that before merge. If unreasonably higher (>10 kcal/mol) the docking pose is discarded.

The process is repeated several time with different docking modes. The best docking complexes poses) according to visual inspection of clashes and energy computation were retained and compared. The poses ranked according to their energies (first priority) and positive interactions of the ligand with the active site.

Molecular Dynamics

Molecular dynamics was performed to check the stability of the complex generated by manual docking according to the following protocol

Length: 1000 fs
Step: 1 fs
Snapshot Every: 5 fs

Supporting Information

Temperature: 300 K
